# Multisensory body representation in autoimmune diseases

**DOI:** 10.1038/srep21074

**Published:** 2016-02-12

**Authors:** Gianluca Finotti, Marcello Costantini

**Affiliations:** 1 Laboratory of Neuropsychology and Cognitive Neuroscience, Department of Neuroscience, Imaging and Clinical Sciences, University G. d’Annunzio, Chieti, Italy & Institute for Advanced Biomedical Technologies - ITAB, University G. d’Annunzio, Chieti, Italy; 2 Centre for Brain Science, Department of Psychology, University of Essex, UK

## Abstract

Body representation has been linked to the processing and integration of multisensory signals. An outstanding example of the pivotal role played by multisensory mechanisms in body representation is the Rubber Hand Illusion (RHI). In this paradigm, multisensory stimulation induces a sense of ownership over a fake limb. Previous work has shown high interindividual differences in the susceptibility to the RHI. The origin of this variability remains largely unknown. Given the tight and bidirectional communication between the brain and the immune system, we predicted that the origin of this variability could be traced, in part, to the immune system’s functioning, which is altered by several clinical conditions, including Coeliac Disease (CD). Consistent with this prediction, we found that the Rubber Hand Illusion is stronger in CD patients as compared to healthy controls. We propose a biochemical mechanism accounting for the dependency of multisensory body representation upon the Immune system. Our finding has direct implications for a range of neurological, psychiatric and immunological conditions where alterations of multisensory integration, body representation and dysfunction of the immune system co-exist.

It is now firmly established that the nervous system is not an immune-privileged environment. Immune signalling is a noticeable feature not only of many pathological brain conditions, but also of normal brain functions. Recent studies, indeed, show that immune molecules of the innate immune system, as well as of the adaptive immune system, are expressed in the nervous system and play important but non-immunological roles in neurodevelopment and synaptic plasticity^1^,^2^. Evidence for the idea that the immune system is involved in normal neurobehavioral processes derive from data showing that cytokines, which are regulators of host responses to infection, are expressed in the healthy brain and that neurons and glia can produce and respond to inflammatory cytokines^3^,^4^. For instance, it has been demonstrated that the secretion of the pro-inflammatory cytokine IL-1β in the hippocampus accompanies long-term potentiation and it is critically involved in maintaining it^5^. On the same vein, chronic exposure to IL-1β produces marked impairments in spatial memory tested with the water maze paradigm, as well as impaired long-term contextual fear memory^6^,^7^. Other pro-inflammatory cytokines play a role in neural functioning. For instance, many studies demonstrated a detrimental effect of elevated IL-6, IL-8, Tumour Necrosis Factor (TNF) and interferon-γ levels on long-term synaptic plasticity^8^ and neural development^9^,^10^.

The pivotal role of immune molecules in normal brain functions allows raising the question as to whether the immune system has a role to play in one of the fundamental abilities of our brain, which is the ability to integrate information from different sensory modalities^11^,^12^,^13^. Specifically, mechanisms of processing and integration of a wide range of multisensory signals are considered crucial in constructing a dynamic representation of the body^11^,^14^,^15^,^16^,^17^,^18^. An outstanding example of the pivotal role played by multisensory mechanisms in body representation is the Rubber Hand Illusion^19^. In this experimental paradigm the simultaneous stroking with two paintbrushes of a rubber hand and the participant’s hidden hand can induce the experience that the rubber hand is their own hand and that it is the rubber hand that “senses” the touch of the paintbrush. Therefore, multisensory integration between vision and touch “changes the phenomenal status of the rubber hand from inanimate object to part of one’s own body”^16^.

The pivotal role of multisensory integration in body representation might bridge the gap between the brain and the immune system. Indeed, despite its role, multisensory integration is not an innate ability. The earliest appearing multisensory neurons in the superior colliculus (SC) are incapable of synthesizing cross-modal stimuli^20^,^21^,^22^. Rather, they require a protracted period of postnatal maturation to develop this capability^23^. Both animal and human studies of the early stages of life show that, during early development, an alteration to the environment or a disruption of processing to one sense can result in a striking degree of neural re-organization between the senses^24^,^25^,^26^,^27^,^28^,^29^. Interestingly, such neural plasticity is deeply intertwined with the immune system and pro-inflammatory cytokines. This claim is supported by a recent study showing that prolonged exposure to pro-inflammatory cytokines causes premature stabilization of developing synapses within the tectum (i.e., the SC in mammals). Therefore, this prevents normal synaptic refinement and elimination that occurs during development. Indeed, during development, the tectal circuitry undergoes a significant amount of refinement, both in the retino-tectal projection, as well as in the local circuitry within the tectum^30^.

Given the detrimental effect of chronic exposure to immune molecules, in particular pro-inflammatory cytokines, in normal brain functions, neurogenesis and synaptic plasticity, we hypothesized that multisensory body representation, as revealed by the rubber hand illusion, is negatively altered in subjects suffering from Coeliac Disease, characterized by chronic exposure to systemic high levels of pro-inflammatory cytokines^31^. The strength of the illusion was measured using proprioceptive drift, defined as the subjective estimate of the distance between the participant’s real hand and the rubber hand, and subjective reports.

## Materials and Methods

### Participants

Nineteen female participants with coeliac disease (CD; mean age 25.7) and twenty-five female control participants (HC; mean age 21.0) were included in the present study. Participants were recruited via public ads. Coeliac cohort was defined as treated coeliac, i.e. adhering to a gluten free diet. All patients had biopsy-proven Coeliac Disease and/or had tissue transglutaminase (TTG) IgA antibody serologic tests performed at the time of diagnosis. The study was approved by the Ethics committee of the “G. d’Annunzio” University, Chieti, and was conducted in accordance with the ethical standards of the 1964 Declaration of Helsinki. All participants were volunteers who gave informed written consent, were free to withdraw at will and were naïve to the rationale of the experiment.

### Experimental design

We used a 2 × 2 × 2 mixed design with order of trial conditions counter-balanced across participants. The viewed object (rubber hand versus piece of wood) and timing of visuo-tactile stimulation (synchronous versus asynchronous) were the within subjects factors. The experimental group (Coeliac Disease and Healthy Controls) was the between subjects factor. The four within-subjects conditions were: (i) rubber hand synchronous; (ii) rubber hand asynchronous; (iii) wood synchronous; and (iv) wood asynchronous. The rubber hand was a realistic prosthetic hand. The piece of wood was a plain wooden block, pale and beige in colour, with a thumb-like feature and with one end tapered into a wrist-like shape. This wooden stimulus had comparable overall size to the rubber hand. Stimulation was delivered manually by the experimenter with the use of two identical paintbrushes. In the synchronous visuo-tactile stimulation conditions, the experimenter manually stroked with two paintbrushes both the participant’s hand and the viewed object at the same time and the same locations. In the asynchronous visuo-tactile conditions, the experimenter stroked the participant’s hand first, while the viewed object was stroked with a latency of 500–1.000 ms in the corresponding location. Each stimulation period lasted 120 s and was timed with a stopwatch.

### Procedure

Participants were welcomed in the laboratory and requested to report their height and weight to calculate body mass index (BMI). Participants were also asked to complete the psychological general well-being index (PGWBI^32^,) questionnaire and the Beck Depression Inventory-II (BDI-II^33^,). BMI, the PGWBI and the BDI-II were recorded to ensure that there were not between-group differences in weight, perceived psychological well-being and depression that could potentially confound the results in the rubber hand illusion. The psychological general well-being index is a validated Health Related Quality of Life (HRQoL) measure, widely used in clinical trials and epidemiological research to provide a general evaluation of self-perceived psychological health and well-being. The Beck Depression Inventory is a 21-question multiple-choice self-report inventory widely used for measuring the severity of depression.

Participants were then exposed to the RHI phase. They sat at a table across from the experimenter with their right hand placed inside a specially constructed box, measuring 100 cm in width, 40 cm in height and 20 cm in depth. The rubber hand rested inside the same box in front of the subject’s midline at the same distance in front of the subject’s chest as the subject’s hand was. The lateral distance between the index finger of the subject’s hand and the index finger of the rubber hand was 27 cm. The entire box was covered by a mirror, which prevented the subject from ever seeing their hand directly. A small half-mirror was set into the mirror just above the rubber hand^34^ (for the experimental setup). Lighting underneath the half-mirror was controlled by the experimenter to make the rubber hand appear (during stimulation) and disappear (during position judgment, see below). We used the proprioceptively perceived position of the subject’s hand as an implicit, quantitative proxy for the illusion. We presented a standard ruler above the table and reflected in the mirror to appear at the same gaze depth as the rubber hand and subject’s hand, and spanning the spatial range between them. Participants were asked, “Where is your index finger”? They responded by verbally reporting a number on the ruler. They were instructed to judge the position of their finger by projecting a parasagittal line from the centre of their index finger to the ruler. During the judgments, there was no tactile stimulation, and subjects were prevented from seeing the rubber hand or any other landmark on the worksurface by switching off the lights under the half-mirror. The ruler was always placed with a different random offset for each judgement to prevent subjects from memorising and repeating responses given on previous trials. Subjects made a baseline judgement before each stimulation trial, and another one afterwards. The difference between these two judgements represented the change in perceived hand position due to the stimulation, and was taken as our measure of RHI. A brief rest period followed each condition. To prevent transfer of the illusion across conditions, the subjects were encouraged to move the hand and body during the rest period. The experimenter then passively replaced the hands in the correct position in preparation for the next trial. After each condition participants were asked to fill in the RHI questionnaire. Finally, Interoceptive Awareness was also measured using the well-established heartbeat detection task^35^. In this task, participants counted the number of heartbeats over separate time periods (25, 35, 45, and 100 seconds) while the experimenter measured the exact/actual count. The researchers then used an algorithm to yield an “accuracy” ratio between 0 (no accuracy) and 1 (perfect accuracy). Interoceptive awareness was measured because previous studies have found a linear relation between susceptibility to the rubber hand illusion and interoceptive awareness, with participants scoring lower in the rubber hand illusion performing better in the heartbeat detection task^36^,^37^.

### Rubber hand illusion questionnaire

We adopted a total of 22 questions from Longo and colleagues^38^ to measure the RHI. The questions referred to four different components of the experience of embodiment during the RHI paradigm: (i) Ten statements referring to the embodiment of the rubber hand. These comprised items relating to the feelings that: the rubber hand belonged to the participant, the participant had control over the rubber hand, the rubber hand and real hand were in the same location, and the rubber hand had taken on features of the actual hand; (ii) six statements referring to the experience of loss of one’s hand. These comprised items relating to the feelings of: being unable to move one’s hand, one’s hand disappearing, and one’s hand being out of one’s control; (iii) three statements referring to the feeling of movement. These comprised items relating to perceived motion of one’s own hand, and to movement of the rubber hand; (iv) three statements referring to affect. These comprised items relating to the appeal and enjoyment of the experience, and the touch being pleasant. Participants completed four versions of the questionnaire, one for each experimental condition. Participants answered each statement by choosing a number from a 7-point Likert Scale, from “ –3 being strongly in disagreement” to “+3 being strongly in agreement”.

### Results BMI, PGWBI and BDI

The mean BMI of the CD group was 22.4 kg/m^2^ (SD 3.3), and for the HC group was 21.3 kg/m^2^ (s.d. 3.4). The difference observed between groups, −1.09, BCa 95% CI [−2.853, 0.976] was not significant (t(42) = 1.1, p = 0.287). The PGWBI global score for the CD group was 70.3 (SD 10.4), and for the HC group was 67.3 (SD 11.7). The observed difference between groups, 3.0, BCa 95% CI [−9.851, 4.537], was not significant (t(39) = 0.85, p = 0.400). Finally, the mean BDI for the CD group was 9.3 (SD 7.6), and for the HC group was 8.3 (SD 6.0). The observed difference between groups, 1.0, BCa 95% CI [−5.475, 3.326], was not significant (t(36) = 0.42, p > 0.677; degrees of freedom are different because PGWBI and BDI-II scores were not available from three and six participants, respectively). The mean Interoceptive awareness was 0.6 (SD 0.2) for both the CD group and the HC group. The observed difference, 0.026, BCa 95% CI [0.097, 0.140], was not significant t(42) = 0.1, p = 0.654.

### Proprioceptive drift

We obtained a baseline pre-test judgment about the subjective location of the participant’s index finger prior to visuo-tactile stimulation and a post-test judgment after stimulation. The pre-test judgments were subtracted from the post- test judgments. The resulting values show the change in the perceived position of the hand between the start and end of the stimulation period, across conditions. We refer to this quantity with the term proprioceptive drift. A positive drift represents a mislocalization of the participant’s hand toward the viewed object (the rubber hand or block of wood), while a negative drift represents a mislocalization of the participant’s hand away from the viewed object. Assumptions of normal distribution, independence of residuals and sphericity were verified. The mean proprioceptive drift per subject for each condition was submitted into a mixed ANOVA with Viewed object (rubber hand versus piece of wood) and the Timing of visuo-tactile stimulation (synchronous versus asynchronous) as within subject factors; and Group (Healthy Controls and Coeliac disease patients) as the between group factor.

**Figure 1:**
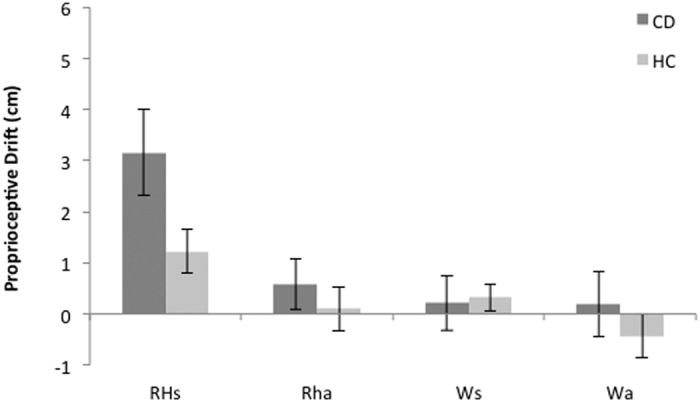
Mean proprioceptive drift and s.e.m. for each group in each experimental condition.

The ANOVA revealed a main effect of viewed object (F(1,42) = 14.5, p < 0.01, η_p_^2^ = 0.25) and timing of visuo-tactile stimulation (F(1,42) = 10.4, p = 0.002; η_p_^2^ = 0.19), as well as an interaction between these two conditions (F(1,42) = 7.2, p = 0.010; η_p_^2^ = 0.147). Given the significant interaction, follow-up t-tests were then conducted. Pairwise statistics showed higher proprioceptive drift in the rubber hand synchronous condition (M = 2.5 cm, SD = 3.0) as compared to all the other experimental conditions (Rubber Hand asynchronous: M = 0.32 cm, SD = 2.12; BCa 95% CI [0.693, 2.886], t(43) = 3.41, p = 0.001, and represented a large effect size d = 1.11; Wood synchronous: M = 0.27 cm, SD = 1.80; BCa 95% CI [1.023, 2.614], t(43) = 3.88, p < 0.001, and represented a large effect size d = 0.99; Wood asynchronous: M = −0.16 cm, SD = 2.41; BCa 95% CI [1. 318, 3.091], t(43) = 4.39, p < 0.001; and represented a large effect size d = 0.91; see Fig. 1).

Importantly, there was also a significant group by viewed object by timing of visuo-tactile stimulation interaction (F(1,42) = 4.3, p = 0.042; η_p_^2^ = 0.09). Greater proprioceptive drift was observed in CD as compared to HC in the rubber hand synchronous condition (observed difference, −1.96 BCa 95% CI [−3.884, −0.229], t(42) = 2.2, p = 0.031; and represented a medium effect size d = 0.67), but not in all the other experimental conditions (see Fig. 1). These differences in proprioceptive drift cannot be explained by differences in perceived baseline location, as the initial error did not differ between groups. The mean proprioceptive mislocalization prior to the induction period was 1.0 cm (SD = 3.6) for the CD group and 2.34 cm (SD = 4.1) for the HC group, and the between-groups difference, 1.28 BCa 95% CI [−0.644, 3.314] was not significant (t(42) = 1.05, p = 0.284). The absence of a significant difference suggests that both the CD and the HC groups had comparable proprioceptive representations prior to the induction period.

### Rubber hand illusion questionnaire

The mean ratings for the four components of the experience of embodiment (Embodiment, Loss of one’s hand, Movement and Affect) were submitted to four separate mixed ANOVAs with viewed object (rubber hand versus piece of wood), the timing of visuo-tactile stimulation (synchronous versus asynchronous) and the components of the illusion as within subject factors, and Group (HC vs CD) as the between group factor. The ANOVA on the embodiment component of the illusion revealed a main effect of viewed object (F(1,42) = 39.1 p < 0.001; η_p_^2^ = 0.48) and timing of visuo-tactile stimulation (F(1,42) = 31.4, p < 0.001; η_p_^2^ = 0.42). The interaction group by viewed object by timing of visuo-tactile stimulation was significant (F(1,42) = 4.4, p = 0.046; η_p_^2^ = 0.09). Paired t-tests indicated that, in both groups, the strength of the RHI was greater during synchronous than asynchronous stimulation (BCa 95% CI [0.694, 0.1.513], t(43) = 4.9, p < 0.001; and it represented a medium effect, d = 0.62). The difference between CD and HC in the embodiment component of the illusion was not significant. Also, the other components of the illusion did not differ across groups.

## Discussion

Given the tight and bidirectional communication between the brain and the immune system we tested the hypothesis that multisensory body representation is altered in patients suffering from Coeliac Disease, an autoimmune disorder characterized by high systemic levels of pro-inflammatory cytokines.

To test our hypothesis we used the rubber hand illusion, a well-known experimental method to investigate multisensory body representation. We found a quantitative stronger illusion in CD patients as compared to healthy controls. In particular, CD patients showed higher proprioceptive drift as compared to HC. The difference in the experience of owning the rubber hand, as measured by the questionnaire, was not significant.

Celiac disease (CD) is an autoimmune disorder of the small intestine caused by a permanent intolerance to gluten proteins in predisposed individuals. In these patients, gluten peptides trigger an abnormal autoimmune response that causes the typical CD tissue lesion characterized by villous atrophy, crypt hyperplasia, and increased numbers of intraepithelial and lamina propria lymphocytes. CD enteropathy is also characterized by elevated systemic level of pro-inflammatory cytokines. For instance, Manavalan and colleagues performed cytokine assays on patients suffering from coeliac disease. They found higher levels of pro-inflammatory cytokines, including interferon-γ, IL–1ß, TNF, IL-6, IL-4, in patients compared to healthy controls. Although the treatment with a gluten-free diet (GFD) usually leads to normalization of mucosal histology, remission of clinical symptoms and decreases levels of systemic pro-inflammatory cytokines^31^,^39^, the available evidence suggests that serum levels of pro-inflammatory cytokines during GFD are still elevated as compared to normal subjects^31^,^40^.

How can we account for altered multisensory body representation in CD patients as compared to healthy controls? The hypothesis we put forward pertains the effect of inflammation on the brain oscillatory activity, which is the neural mechanism enabling multisensory integration^41^.

An increasing amount of evidence is accumulating supporting the idea that oscillatory activity of the brain, far from being mere noise, represents an instrument that can be used in sensory processing^42^,^43^, and multisensory integration. This is the case in at least two well-known multisensory illusions, namely the double-flash illusion (DFI^44^ and the McGurk illusion^45^). The double-flash illusion is a multisensory illusion where one briefly presented visual stimulus is accompanied by two rapidly presented either auditory (audio-visual DFI) or tactile stimuli (visuo-tactile DFI). Both paradigms frequently induce the perception of a second, illusory visual stimulus. The DFI is optimally perceived if the visual stimulus is presented between the two auditory or tactile stimuli and if all stimuli are presented within ~100 ms^46^. This window of integration is, nevertheless, variable across individuals and seems to be related to the individual’s alpha frequency peak^47^. In a recent MEG study, Lange *et al.*^48^ investigated how fluctuations of prestimulus neuronal activity influence perception of the visuo-tactile DFI. The authors found that the illusory perception of a second visual stimulus was preceded by a reduction of prestimulus alpha power in visual cortex relative to the perception of one visual stimulus. Hence, decreased prestimulus alpha power correlated with the conscious perception of two stimuli.

Recently, Cecere and colleagues^47^ provided causal evidence of a link between brain oscillatory activity, especially in the alpha frequency band, and audio-visual DFI. They firstly recorded EEG while participants performed the audio-visual DFI task and found positive correlation between individual’s alpha frequency peak and the size of the temporal window of the illusion. Participants then performed the same task while receiving occipital transcranial alternating current stimulation (tACS), to modulate individual alpha frequency peak. Results showed that experimentally increasing or decreasing individual alpha frequency peak enlarged and shrunk the temporal window of illusion, respectively. This empirical evidence strongly supports the view that different temporal features of the brain oscillatory activity can predict processing of multisensory stimuli.

Interestingly, high levels of pro-inflammatory cytokines, as in the case of CD patients, change brain oscillatory activity^49^. Such a change is likely to reflect alteration of the excitation/inhibition balance, which is contributed by GABAergic interneurons^50^. Empirical studies have indeed shown that pro-inflammatory cytokines increase the protein expression of GABA transporter type 1 and 3, which are the two important subtypes of GATs responsible for the regulation of extracellular GABA levels in the brain. In particular, GAT1 transporter removes GABA from the synaptic cleft^51^, while GAT3 mediates uptake of GABA from the synaptic cleft by surrounding glial cells^52^. Overall, the removal of GABA from the synaptic cleft is likely to decrease the inhibitory effect of GABA, altering neural oscillatory activity^53^.

If our argument is at stake, one may formulate interesting hypotheses on the effect of inflammation on multisensory processing. For instance it could be hypothesized that participants with ongoing inflammation show altered temporal window of integration, as measured with a simultaneity judgment task^54^, and altered perception of the double flash illusion.

Despite this evidence, it must be noticed that the role of oscillatory activity in multisensory integration is still matter of debate. Classically, convergence of unimodal inputs in multisensory areas has been regarded as the primary mechanism for multisensory integration. According to this view, multisensory integration results from a linear integration of unimodal stimuli, reflected in a change of firing frequency in multisensory neural populations^55^. Although the important role of multisensory integration is largely established, the mechanisms underlying multisensory integration are not yet fully understood. If neural oscillations seem to provide a more flexible model (see^56^) it is clear that it could be not the only mechanism responsible for multisensory integration. For instance, it might be argued that altered stimulus-driven neural response, rather than altered oscillatory activity in CD patients might account for our results. Such an explanation is not at odds with our proposal. Stimulus-driven neural response and oscillatory activity are tightly linked to each other, so that the former depends on the latter at the time the stimulus impinges on brain oscillatory activity^57^. For instance, Scheeringa and colleagues (2011), using simultaneous electroencephalography as a measure of ongoing activity and functional magnetic resonance imaging (fMRI) as a measure of the stimulus-driven neural response, investigated whether the phase of occipital alpha oscillations at the onset of a visual stimulus affected the amplitude of the visually evoked fMRI response. Results showed that stimuli arriving at the peak of the alpha cycle yielded a lower blood oxygenation level-dependent (BOLD) fMRI response in early visual cortex than stimuli presented at the trough of the cycle^57^.

Another finding that deserves to be discussed is the dissociation we observed between proprioceptive drift and subjective report. Indeed, while CD patients showed higher proprioceptive drift as compared to HC, subjective report did not differ between the two groups. The rubber hand illusion is thought to be the product of the three-way interaction between vision, touch and proprioception^19^. Several findings suggest that proprioceptive drift depends upon multisensory integration, while the illusory ownership involves a second mechanism of comparison with internal models of the body (for a review see^58^,^59^). Our hypothesis of the impact of inflammation on multisensory integration fits with this finding, and supports the dissociation sometimes observed between proprioceptive drift and subjective report of the RHI^60^,^61^.

Whilst results are intriguing and foster a cross-disciplinary work we only provide correlational, rather than causal, links between multisensory integration and inflammation. Moreover, a limitation of our study must be taken into account. We did not measure the level of systemic pro-inflammatory cytokines in our participants at the time of testing, thus, the link between inflammation and Coeliac disease is based on the current literature. This also made not possible to test whether patients with more severe inflammation showed higher sensitivity to the RHI. This hypothesis would be particularly interesting to investigate as there is already available evidence in literature of a link between the IS and the RHI. For instance Barnsley and colleagues found that the rubber hand illusion increases histamine reactivity in the real arm of participants^62^. Despite our patients performed clinical tests at the time of diagnosis, the most widely used system of classification of coeliac disease severity, the Marsh-Oberhiber classification^63^, could not be used here as an indicator of severity of inflammation. This system categorizes histological modifications, and thus it cannot be used to this end^63^. What is more, despite coeliac patients have higher systemic levels of peripheral pro-inflammatory cytokines, the peripheral cytokine profile does not mirror the cytokine profile of the inflamed small intestine mucosa^31^. Still, the possibility that higher levels of pro-inflammatory cytokines at the time of testing would result in altered multisensory integration is a compelling one and needs to be further investigated in future research.

## Additional Information

**How to cite this article**: Finotti, G. and Costantini, M. Multisensory body representation in autoimmune diseases. *Sci. Rep.* **6**, 21074; doi: 10.1038/srep21074 (2016).
